# Trends in the Distribution of Gestational Age and Contribution of Planned Births in New South Wales, Australia

**DOI:** 10.1371/journal.pone.0056238

**Published:** 2013-02-20

**Authors:** Natasha Nassar, Michal Schiff, Christine L. Roberts

**Affiliations:** 1 Clinical and Population Perinatal Research, Kolling Institute of Medical Research, University of Sydney, New South Wales, Australia; 2 Department of Obstetrics, Gynaecology, and Neonatology, University of Sydney at Royal North Shore Hospital, St Leonards, New South Wales, Australia; UCL Institute of Child Health, University College London, United Kingdom

## Abstract

**Background:**

There is concern that the rate of planned births (by pre-labour caesarean section or induction of labour) is increasing and that the gestation at which they are being conducted is decreasing. The aim of this study was to describe trends in the distribution of gestational age, and assess the contribution of planned birth to any such changes.

**Methods:**

We utilised the New South Wales (NSW) Perinatal Data Collection to undertake a population-based study of all births in NSW, Australia 1994–2009. Trends in gestational age were determined by year, labour onset and plurality of birth.

**Results:**

From 1994–2009, there was a gradual and steady left-shift in overall distribution of gestational age at birth, with a decline in the modal gestational age from 40 to 39 weeks. For singletons, there was a steady but significant reduction in the proportion of spontaneous births. Labour inductions increased in the proportion performed, with a gradual and changing shift in the distribution from a majority at 40 weeks to an increase at both 37–39 weeks and 41 weeks gestation. The proportion of pre-labour caesareans also increased steadily at each gestational age and doubled since 1994, with most performed at 39 weeks in 2009 compared with 38 weeks up to 2001.

**Conclusions:**

Findings suggest a changing pattern towards births at earlier gestations, fewer births commencing spontaneously and increasing planned births. Factors associated with changing clinical practice and long-term implications on the health and well-being of mothers and babies should be assessed.

## Introduction

Duration of pregnancy, which is reported as the number of completed weeks of gestation, is the leading perinatal predictor of health outcomes in babies. [1] Fetal growth and development is a continuum and not complete until around the *due date of birth,* at 40 completed weeks gestation. [2] However, most studies tend to assess gestation as a binary factor in the form of preterm, <37 completed weeks’ gestation versus term, 37–41 weeks, and potentially masking any effect across the gestational age range. It is well established that infants born preterm are at increased risk of severe neonatal morbidity and death.[3]–[5] However, most studies have focused on those born very preterm <32 weeks gestation, with recent evidence suggesting that infants born late preterm at 34–36 weeks’ and early term at 37–38 weeks’ gestation may also be at greater risk for adverse complications compared with infants born at term, 39–41 weeks’. [6], [7] Monitoring trends in the gestational age distribution can, thus, be an important indicator of long term health and developmental outcomes.

Gestational age at birth is conditional on either spontaneous onset of labour or a clinical decision that birth should occur. Planned (also referred to as elective or iatrogenic) births, by induction of labour or pre-labour caesarean section follows a considered decision about continuing the pregnancy that balances the potential benefits and harms of early delivery for the mother and baby. [8] Decisions relating to the gestational week at which planned births are conducted have important health implications. Planned births are reported to be increasing internationally, [9], [10] and the impact on neonatal and maternal morbidity varies dependent on the gestational age studied.[10]–[15] Improvements in neonatal care and survival may be one of the factors influencing changes in timing around birth. Most previous studies have focused on outcomes following late preterm birth (32–36 weeks), however their findings have been mixed with some reporting reduced, [16], [17] and others improved perinatal survival and health. [14], [15], [18] More recent contention has been around planned births conducted at early term and before 39 weeks, however there is less evidence surrounding its effectiveness and safety. Nevertheless, studies suggest increased neonatal morbidity amongst these infants compared to those born at 39 weeks or greater.[11]–[13] To date, there has been no assessment of the trend in gestational age at birth and the role of planned births. The aim of this study was to describe population trends in the distribution of gestational age, and assess the contribution of planned birth to any such changes in the distribution.

## Methods

### Study Population and Data Sources

The study population included all births of at least 20 weeks gestation in New South Wales (NSW), Australia from January 1994 to December 2009. Data were obtained from the NSW Perinatal Data Collection (PDC), a statutory population-based surveillance system covering all livebirths and stillbirths in NSW of at least 20 weeks gestation or 400 g birth weight. Information is recorded by either the midwife or medical practitioner attending the birth, and includes demographic, medical and obstetric information on the mother, as well as details of labour, delivery, and condition of the neonate. Only de-identified data were provided to researchers. The study was approved by the NSW Population and Health Services Research Ethics Committee.

In the PDC, gestational age is reported as the number of completed weeks of pregnancy. It is determined by the best clinical estimate including early ultrasound (>97%) and the first day of last menstrual period. Onset of labour (spontaneous, induction or pre-labour caesarean section) and plurality of birth (singletons versus multiples) are reported by check-box. Other maternal characteristics and birth outcomes assessed were maternal age (<20, 20–34 and 35+ years), parity (first versus subsequent births), maternal country of birth (Australian versus non-Australian-born), giving birth in a public or private hospital, pregnancy complications (pre-existing diabetes mellitus, gestational diabetes, chronic hypertension, pregnancy-induced hypertension or preeclampsia), preterm birth (<37 weeks’ gestational age), birthweight percentile [19] and stillbirth. Compared with medical records, the information collected on the PDC has been demonstrated to be reliable, with high levels of agreement and low rates of missing data. [20].

### Analysis

Of 1,427,527 births, records missing a gestational age (n = 378), labour onset (n = 390), or plurality of birth (n = 58) were excluded, leaving 1,426,701 (99.9%) records available for analysis. Trends in maternal characteristics and birth outcomes were examined using Cochrane-Armitage test and review of relative changes over time. Changes in the onset of labour between 1994 and 2009 were investigated by assessing the percentage of spontaneous births, labour inductions, or pre-labour caesareans among all births occurring in each year. Onset of labour was examined separately for singleton and multiple pregnancies.

Annual distribution in gestational age throughout the study period were determined with the proportion of births in each week of gestation calculated (number of births occurring in a specific gestational week per total births which took place that year). Analysis was also carried out for each category of labour onset and stratified by singletons and multiples (as denominators) to show the relative contribution of each group. As relatively small number of records of multiple pregnancies resulted in greater annual fluctuations, analysis was carried out by grouping data into four-year intervals. To account for changes in pregnancy risk factors over time, sensitivity analyses were conducted among low risk primiparae (women having a first birth), aged 20–34 years, without pregnancy complications (defined above), and with a liveborn singleton infant, born in cephalic presentation and of normal fetal growth at the 10^th^–90^th^ birth weight percentile of the distribution for gestational age and infant sex. [21].

## Results

From 1994 to 2009, the annual number of women giving birth in NSW increased by 10% (Table 1). In parallel, median maternal age increased (29 years in 1994 versus 31 years in 2009) as a result of an increasing proportion of women aged 35 years or more (13% vs. 24%). Multiple birth rates remained relatively constant, women were less likely to be classified as low risk (59% vs. 51%) and more likely to be having a first birth (40% vs. 42%); give birth in a private hospital (15% vs. 25%) and deliver preterm (5.9% vs. 6.6%) (test-for-trend for all factors, P<0.01). These changes were accompanied by 27% and 88% increases in the rates of labour induction and pre-labour caesarean section, respectively. Stillbirth rates did not change significantly (0.56/100 versus 0.62/100 births, trend p = 0.07).

**Table 1:** Maternal characteristics and birth outcomes and relative change throughout the study period, 1994–2009.

	1994 N (%)	1999 N (%)	2004 N (%)	2009 N (%)	Relative change§(%)
**All mothers**	**86,463**	**85,961**	**84,283**	**95,033**	**9.9**
Maternal age (years)					
<20	4,358 (5.1)	4,098 (4.8)	3,387 (4.0)	3,292 (3.5)	−31.4
20–34	70,680 (81.8)	67,167 (78.2)	64,110 (76.1)	69,068 (72.7)	−11.1
35+	11,313 (13.1)	14,668 (17.1)	16,769 (19.9)	22,658 (23.8)	81.7
Nulliparous	34,194 (39.5)	35,308 (41.1)	35,795 (42.5)	40,345 (42.4)	7.3
Non-Australian born	22,587 (25.9)	23,728 (27.2)	23,471 (27.5)	30,278 (31.5)	21.6
Multiple birth	1,212 (1.4)	1,291 (1.5)	1,305 (1.5)	1,366 (1.4)	0
Low risk women†	51,084 (59.1)	47,018 (54.7)	45,313 (53.8)	48,286 (50.8)	−14.0
Preterm birth	5,067 (5.9)	5,562 (6.5)	5,553 (6.6)	6,302 (6.6)	11.9
Private hospital	13,357 (15.4)	15,869 (18.5)	20,551 (24.4)	23,478 (24.7)	60.4
Labour induction	17,536 (20.3)	20,612 (24.0)	20,549 (24.4)	24,473 (25.7)	26.6
Pre-labour caesarean section	8,055 (9.3)	9,147 (10.6)	12,929 (15.3)	16,649 (17.5)	88.2
**All babies** *	**87,705**	**87,283**	**85,621**	**96,429**	**9.9**
Livebirths	86,980 (99.2)	86,734 (99.4)	85,056 (99.3)	95,827 (99.4)	0.2
Stillbirths	494 (0.56)	533 (0.61)	561 (0.66)	598 (0.62)	10.1

§ Relative change was calculated by: [(2009 rate –1994 rate)/(1994 rate)].

* 100; Test-for-trend was significant for all factors except stillbirths and multiple births, P<0.001.

† Low risk pregnancies defined as primiparae, aged 20–34 years, without pregnancy complications; and with a liveborn singleton infant, born in cephalic presentation and of normal fetal growth at the 10^th^–90th birth weight percentile of the distribution for gestational age and infant sex. [16].

* Numbers may not add up to totals due to missing data or rounding.

**Figure 1:**
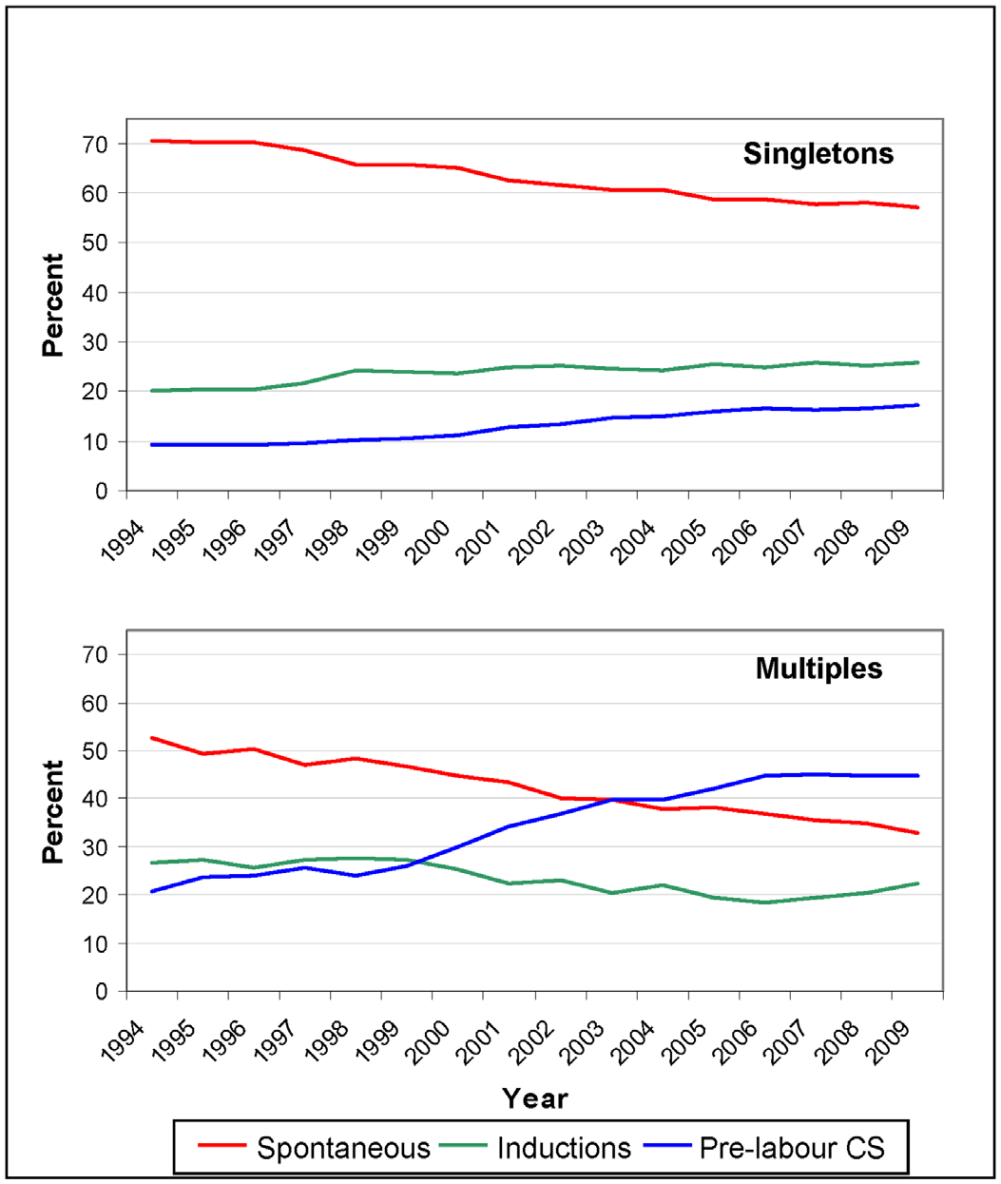
Trends in onset of labour in singleton (top) and multiple (bottom) births, 1994–2009.

Among singleton pregnancies, a decreasing trend in spontaneous labour was offset by an increase in pre-labour caesareans and labour inductions (Figure 1). Between 1994 and 2009, the rate of caesareans for multiple births more than doubled from 20% to 45%, while the rate of spontaneous births and inductions declined from 53% to 33% and 27% to 22%, respectively (Figure 1).

**Figure 2:**
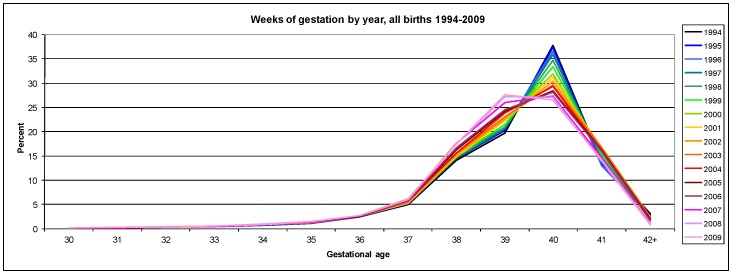
Distribution of gestational age at birth NSW, Australia, 1994–2009.

During the study period, a steady left-shift towards earlier gestations was observed in the overall distribution of gestational age at birth (Figure 2). Observed trends included a gradual and continuous decline in the frequency of births at 40 weeks’ gestation offset by a corresponding increase in all births at 39 weeks. By 2008 the modal gestational age had decreased to 39 weeks gestation. There was a steady increase in the annual proportion of births occurring at each week from 33 to 39 weeks’ gestation, while births 42 weeks’ or greater declined from 3.2% in 1994 to only 0.8% in 2009.

**Figure 3:**
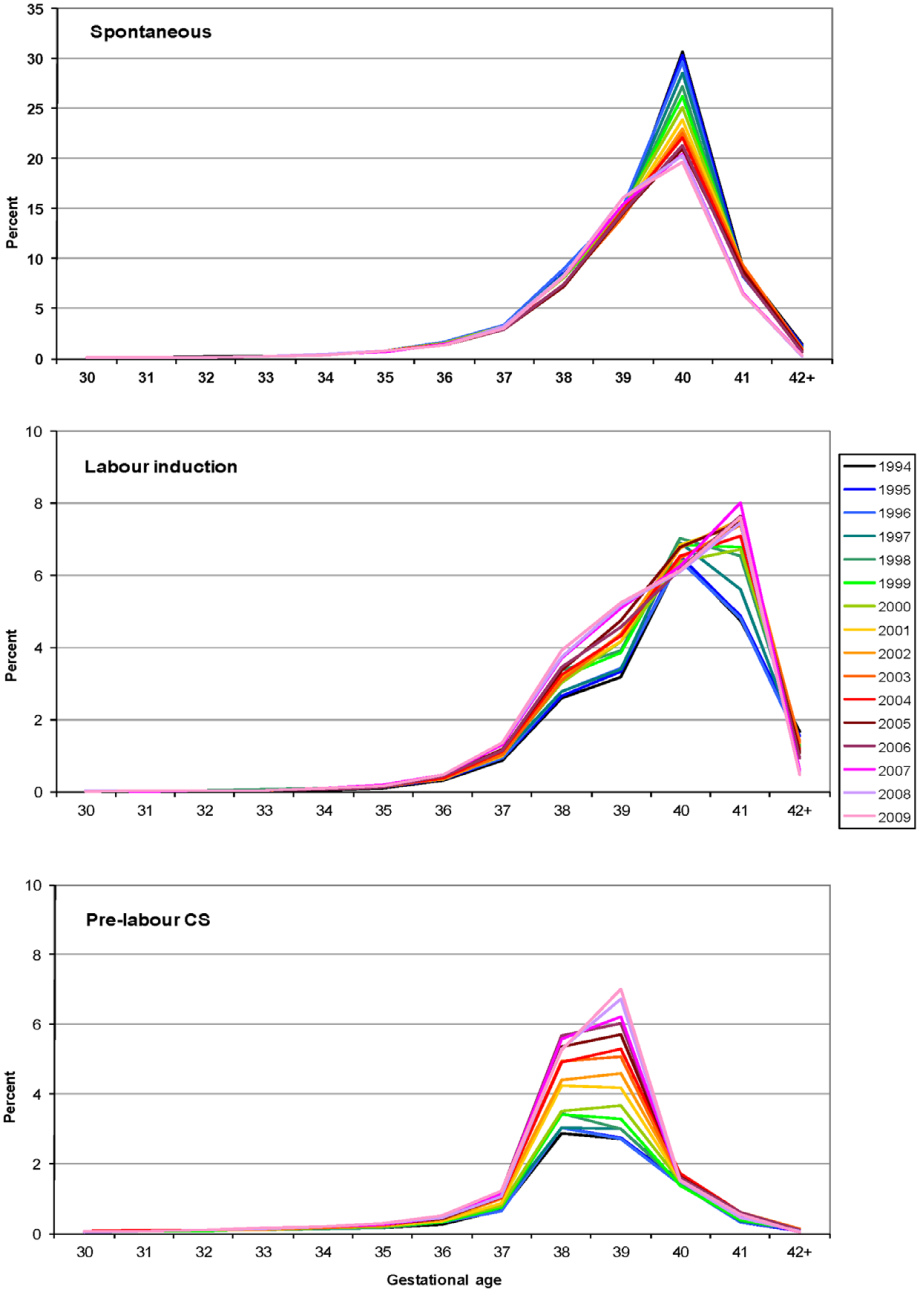
Distribution of gestational age at birth for singleton births by onset of labour, 1994–2009. Spontaneous labour (top), induced labour (middle) and pre-labour caesarean section (bottom).

A similar shift was observed when singleton births were investigated separately. However, different patterns were revealed when examined by labour onset (Figure 3A–C). For singleton births with spontaneous labour, 40 weeks gestation remained the modal gestation but the proportion decreased markedly from 31% of all singleton births in 1994 to only 20% in 2009. In addition, from 2007 there was a left-shift due to increased spontaneous onset of labour, primarily at 37–39 weeks (Figure 3A). However, from 40 weeks’ gestation there was a decrease in the proportion of births. Also, of note, spontaneous preterm births (<37 weeks) decreased from 3.7% in 1994 to 3.3% in 2009 (trend p<0.01). The distribution of planned births among singleton pregnancies also changed over time. For labour inductions the modal gestational age shifted from 40 to 41 weeks gestation with the increase at 41 weeks accompanied by a marked and continuing increase in the proportion of inductions also performed at 37–39 weeks (Figure 3B). There was also a large increase in the contribution of pre-labour caesarean section to all singleton births, almost doubling since 1994 from 9.1% to 17.1%. Pre-labour caesareans at 38–39 weeks increased steadily from 1997, with caesareans performed at 39 weeks gestation surpassing those at 38 weeks from 2001 (Figure 3C). The resulting shift of the peak of pre-labour caesarean sections from 38 to 39 weeks further intensified from 2008. When analyses were restricted to low risk primiparae with singleton pregnancies, observed trends in gestational age resembled those of all singletons (data not shown).

**Figure 4:**
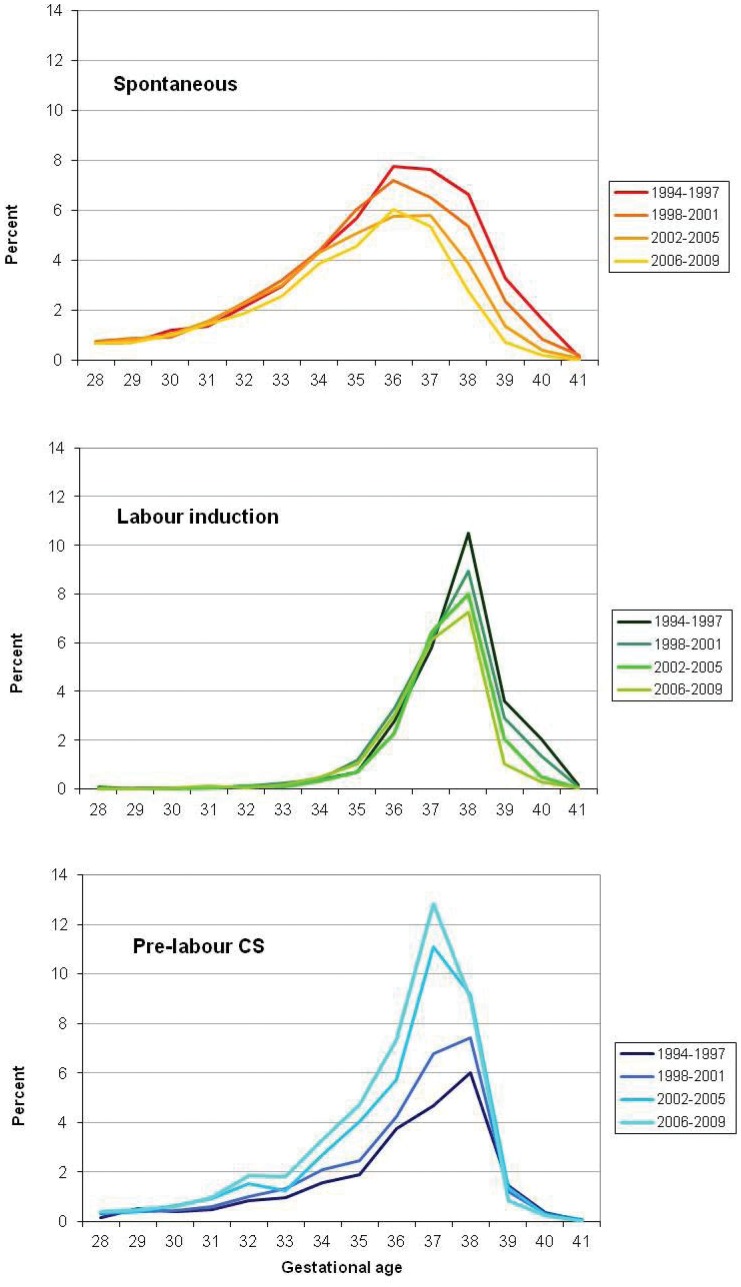
Distribution of gestational age at birth for multiple births by onset of labour, 1994–2009. Spontaneous labour (top), induced labour (middle) and pre-labour caesarean section (bottom).

Among multiple births, the modal gestational age decreased from 38 weeks gestation in the 1990’s to 37 weeks from 2003 onwards (data not shown). Again the trend differed by labour onset (Figure 4A–C). The modal gestation for spontaneous labour was generally 36 weeks although declining from 7.7% of all multiple pregnancies in 1994–1997 to 6.0% in 2006–2009. A substantial and continuous decline was observed in spontaneous labour at ≥37 weeks but also at 32–35 weeks from 2006 (Figure 4A). The peak of inductions remained at 38 weeks gestation, with the proportion ≥38 weeks decreasing steadily and those at 37 weeks subtly increasing (Figure 4B). There was a marked increase in the contribution of pre-labour caesarean sections <39 weeks (Figure 4C) with the modal gestation for pre-labour caesareans shifting from 38 to 37 weeks.

## Discussion

We report a gradual decreasing trend in the gestational age of babies born in NSW, Australia, between 1994 and 2009. The most common gestational age has changed from 40 to 39 weeks, with early term births (37–38 weeks) becoming more frequent. The observed trends occurred among both singleton and multiple pregnancies, with findings revealing a steady and constant reduction in spontaneous births and increasing planned births, particularly pre-labour caesareans. Despite the rise in obstetric interventions over the study period, a corresponding reduction in fetal mortality has not been reported in our New South Wales population. [20] However, the converse argument that the lack of a rise in fetal demise may be due to the increase in planned births, cannot be excluded. These changes in gestational age and timing of birth may be due to increased survival and advances in neonatal care. Further, outcomes of infants born close to or at early term may be presumed to be similar to those born at 40 weeks gestation, particularly if they look similar and appear physiologically and developmentally mature. This is of some concern as evidence suggests impact of planned births may be related to gestational age. Studies of planned births for late preterm infants have revealed conflicting results, with some showing reduced [16], [17] and others improved perinatal survival and health; [14], [15], [18] while early term births have been found to be associated with increased infant and maternal morbidity.[11]–[13].

Similar findings were reported by Davidoff et al, 2006 who identified a left shift in gestational age distribution in both spontaneous births and those associated with planned births among singleton births in the US from 1992 to 2002. [22] One of the differences between our study and the Davidoff work is that the changes in gestational age we report for planned births remain within the limit of term pregnancies with an increase in the proportion of early term births at 37–39 weeks gestation, paralleled by a decrease on the latter side of term at 40–42 weeks. In contrast, the US study reports an additional marked increase in the rates of late preterm births at 34–36 weeks’. [22] Differences in our Australian results, including the shift in the peak of pre-labour caesarean sections among singleton births from 38 to 39 weeks in recent years since 2008 may be attributable to recent introduction of the state-based maternity care policy targeting timing of pre-labour caesarean section. [23].

Findings highlight that not only has the rate of planned births increased significantly, but the gestation at which they are being conducted is decreasing. By 2009, almost half of all births were iatrogenically timed and almost one in four singleton infants were delivered before the due delivery date, 13% by pre-labour caesarean and 10% by labour induction. [11] The increase in planned births was observed at all gestational ages up to 40 weeks and median gestation for this group decreased to 39 weeks. Although planned birth is of demonstrated benefit amongst selected high-risk pregnancies (eg. women with hypertensive disease), for many conditions (eg diabetes, growth restriction, preterm pre-labour rupture of membranes) evidence is lacking. Of particular concern, are recent studies which report up to half of all planned births at term are performed in low risk women [11] and in many cases for unspecified reasons. [11], [24], [25] The changing pattern towards fewer births commencing spontaneously and increased use of caesarean section has in itself potential adverse health consequences for both mothers and infants. For mothers, caesarean section, particularly pre-labour have been associated with greater complications in subsequent pregnancies, including uterine rupture and placental implantation problems, and longer maternal hospital stays.[11], [26]–[29] For infants, vaginal birth has been shown to be important for fetal lung and immune system development. [30], [31] Long-term morbidities of term infants such as asthma, hay fever, diarrhoea, respiratory & food allergies have been associated with birth by pre-labour caesarean section. [6], [32], [33] However, in light of inconsistent results, further adequately powered long-term studies taking into account timing of delivery are required to confirm or refute these findings.

Despite no change in the prevalence of multiple births between 1994 and 2009, there was a decrease in modal gestational age for multiples from 38 to 37 weeks’ with changes in the gestational age distribution larger and different to singleton births, especially by onset of labour. Of note, we identified a left shift in spontaneous births and pre-labour caesarean section, and inductions at 38 weeks or more decreased steadily. These changes, particularly among planned births have occurred since 2002, although there has been no change in the evidence regarding delivery management for twins; with two trials currently underway. [34], [35] This highlights the importance of assessing and reporting changes in gestational age, separately, by plurality. However, given multiple births only contribute a small proportion of all births, changes in distribution of gestational age appears to be driven predominately by potential changes in the management of singleton births.

The trend towards delivery at earlier gestational ages has been reported internationally. [15] It is likely that increased survival and perceived low morbidity risk has influenced obstetric decision-making in lowering the threshold at which delivery is considered. Reviews supporting labour induction for prolonged pregnancy [36] and those showing increased maternal risk in vaginal birth after caesarean [37], [38] may have contributed to this change in clinical attitudes. In addition, obstetric care providers may time pre-labour caesarean or labour induction to balance patient-care responsibilities, accommodate patients’ schedules or avoid the presumed legal risk of expectant management. [39], [40] Increased medical surveillance and monitoring may also lead to more delivery interventions and it has been posited that the risk tolerance for abandoning vaginal delivery and the threshold for obstetric intervention has decreased with advances in neonatal care. [6], [39], [40] We, and others, have found changes in maternal demographic, pregnancy factors or medical risk profiles explain little of the rising rates of planned births. [11], [16], [41], [42] Our findings of similar patterns among low risk women suggest that changes in risk or indication have not explained rising rates of pre-labour caesareans or inductions.

The strength of this study is the use of population-based, prospective and routinely collected data over a 16-year period. Coverage of all births in NSW, the most populous state of Australia, comprising over one third of the nation’s births, [5] also ensures generalisability of the findings. However, it must be noted that we were limited by lack of data relating to obesity and additional medical conditions (other than diabetes and hypertension), which may be indications for planned births. The other potential limitations of the study are related to estimation of gestational age. Previous studies have consistently shown that estimates of gestation based on results from first trimester ultrasound shifts the gestational age distribution towards earlier gestations relative to that based on the last menstrual period. [43], [44] Consequently, early ultrasound estimation of gestational age may lead to higher rates of preterm birth compared with estimates based on the last menstrual period.[43]–[45] Although increased use of early ultrasound may partially explain the observed shift in gestational age to the left, the fact that it was already widely used in the early 1990’s, [46] it is unlikely that changes in classification of gestational age solely account for our findings.

In summary, findings suggest a changing pattern towards births at earlier gestations, fewer births commencing spontaneously and increasing planned births. Factors associated with changing clinical practice and long-term implications on the health and well-being of mothers and babies should be assessed.
